# Pancreatic Cancer Patients Supportive Care Needs: A Qualitative Analysis

**DOI:** 10.1002/pon.70135

**Published:** 2025-03-23

**Authors:** Sara E. Fleszar‐Pavlovic, Roberto M. Benzo, Rui Gong, Amber Browder, Aria Nawab, Arianna E. Brito, Nipun B. Merchant, Frank J. Penedo

**Affiliations:** ^1^ Sylvester Comprehensive Cancer Center University of Miami Miami Florida USA; ^2^ The Ohio State University Comprehensive Cancer Center Columbus Ohio USA; ^3^ Division of Cancer Prevention and Control Department of Internal Medicine College of Medicine The Ohio State University Columbus Ohio USA; ^4^ Division of Medical Oncology Department of Medicine Miller School of Medicine University of Miami Miami Florida USA; ^5^ Department of Surgery University of Miami Miller School of Medicine Miami Florida USA; ^6^ Department of Psychology University of Miami Miami Florida USA

**Keywords:** cancer, oncology, pancreatic neoplasms, psychosocial support systems, qualitative research, quality of life, supportive care needs

## Abstract

**Objective:**

Pancreatic cancer (PaCa) patients face a 5‐year survival rate of just 13%. Most patients present with unresectable disease and endure aggressive treatments with significant chronic and debilitating side effects. PaCa patients also experience significant unmet supportive care needs (e.g., physical, psychological, informational/educational); however, limited qualitative studies have explored the specific needs of racially and ethnically diverse PaCa populations.

**Aims:**

This study identified supportive care needs in a racially and ethnically diverse sample of PaCa survivors.

**Methods:**

As part of a larger project to develop a psychosocial symptom management intervention, semi‐structured qualitative interviews were conducted with PaCa survivors to explore the supportive care needs at diagnosis and after treatment. Qualitative data were analyzed using Rapid Qualitative Analysis, and personal/medical characteristics were analyzed using descriptive statistics.

**Results:**

PaCa survivors (*n* = 18; *M*_age_ = 64) participated, with the majority identifying as female (66.7%), White (88.9%), and Hispanic (55.6%). Over one‐third completed interviews in Spanish. Four themes emerged: (1) information/health system needs, including difficulty understanding complex medical concepts, limited holistic care, post‐treatment symptom management, and health behaviors; (2) psychosocial needs related to quality of life and relationships with family and healthcare providers; (3) physical and functional needs, including persistent side effects and lifestyle changes; and (4) positivity and gratitude.

**Conclusions:**

We emphasize the themes of unmet supportive care needs in a racially and ethnically diverse sample of PaCa survivors. These findings underscore the importance of developing interventions to address these gaps and improve the overall quality of life for diverse PaCa patients.

## 1 Background

Pancreatic cancer (PaCa) is the third leading cause of cancer‐related death in the US, with a 5‐year overall survival of 13% [1]. At diagnosis, most tumors are unresectable due to advanced disease or distant metastases. As a result, only 15%–20% of patients are candidates for potentially curative surgery [2, 3]. The advanced stage of presentation often requires the use of aggressive chemo‐ and radiation therapies, which are associated with severe and sometimes persistent physical side effects (e.g., fatigue, poor appetite, gastrointestinal dysfunction) [4, 5, 6]. The primary surgical option for many PaCa patients is a pancreaticoduodenectomy (i.e., Whipple procedure), a complex procedure that results in significant post‐operative complications (e.g., infection, intra‐abdominal bleeding) for 35%–50% of patients [7, 8, 9, 10]. Comprehensive treatments that include chemotherapy (neo‐and/or post‐adjuvant) and/or radiation and surgery lead to short‐ and long‐term symptom burden, toxicities, and functional declines such as gastrointestinal functional changes, diminished exercise capacity, nausea, pain, and fatigue, and decreased quality of life (QOL), including poor social and emotional well‐being [11, 12, 13, 14, 15, 16]. As a disease that occurs primarily in those > 70 years old [17], age‐related comorbidities, functional limitations, polypharmacy, and other stressors such as social isolation and financial challenges can further exacerbate compromises in QOL [18, 19, 20, 21]. Further, underrepresented populations (e.g., African American/Black and Hispanic populations) with PaCa are more likely to be diagnosed at later stages, receive treatment at low‐volume hospitals, and have lower rates of surgical resection, chemotherapy, and radiation compared to other demographic groups, contributing to higher morbidity and mortality rates [22, 23, 24].

Fitch's [25] Supportive Care Framework highlights the importance of a comprehensive approach to addressing cancer patients' needs across the entire disease trajectory. This framework identifies seven key domains of supportive care (i.e., informational, emotional, practical, physical, psychological, social, and spiritual needs) that should be assessed to optimize patient outcomes. Patients with PaCa often experience substantial unmet supportive care needs, particularly within the informational, physical, and psychological domains, with about half of patients reporting moderate‐to‐high levels of unmet needs [26]. Within the informational domain, nearly one‐third of patients felt that the information received at diagnosis was insufficient, and many expressed a desire for greater involvement in treatment decisions [27]. Common unmet physical needs such as pain and fatigue are closely linked with heightened anxiety and depression in patients with PaCa [18, 27]. These unmet needs are especially acute in patients with unresectable disease, where psychological distress and daily living challenges are most severe [18]. Gooden and White [28] identify gaps in managing pancreatic exocrine insufficiency (PEI), a condition in which the pancreas fails to produce sufficient enzymes leading to poor nutritional absorption. The PEI management gaps—stemming from inadequate dietary consultation, patient education, enzyme supplement prescribing, and understanding of enzyme replacement therapy—significantly contribute to poor quality of life.

While prior studies have documented the complex and evolving supportive care needs of PaCa patients, most rely on survey‐based quantitative assessments that may not fully capture the nuances of patients' lived experiences. These studies often utilize predefined categories that limit the ability to express specific concerns and are predominantly conducted in homogeneous populations outside the U.S. As the 5‐year survival rate steadily increases (the survival rate has reached 13%, marking a consistent rise for the third consecutive year); (1) and treatment options continue to expand, it is crucial to understand and anticipate the full spectrum of supportive care needs for PaCa patients. Identifying and addressing these needs should be initiated early for all patients diagnosed with PaCa to improve their quality of life throughout treatment and beyond. Although previous literature captures the complex supportive care needs of PaCa patients, qualitative research on these needs remains limited, particularly among racially and ethnically diverse populations. To address this gap, we conducted a qualitative study to explore the supportive care needs of a diverse sample of PaCa survivors in Miami.

## 2 Methods

### 2.1 Sampling and Recruitment

PaCa survivors (*N* = 18) participated in one‐on‐one semi‐structured interviews to identify specific supportive care needs during their treatment for PaCa. The current study was part of a larger study aiming to develop and examine the feasibility and acceptability of a psychosocial symptom management intervention in PaCa patients (clincialtrials.gov #04815746). Eligible PaCa survivors were (1) ≥ 18 years old, (2) previously treated for pancreatic neoplasms or neuroendocrine tumors at Sylvester Comprehensive Cancer Center (SCCC) at the University of Miami Miller School of Medicine (UM); and (3) fluent in English or Spanish. Patients with severe mental illness or overt signs of severe psychopathology within the past 6 months were excluded.

Potential participants were recruited via various methods: (1) the UM's consent‐to‐contact database, where patients voluntarily enroll and consent to be contacted about clinical study opportunities; (2) clinician‐based referrals; and (3) self‐recruitment (i.e., community advocates, support groups, and flyers). A study team member contacted potential participants via telephone to introduce the study and confirm eligibility. Participants who consented and enrolled in the study were invited to complete one‐on‐one interviews.

### 2.2 Data Collection

One‐on‐one interviews were offered both in‐person and via Zoom, depending on participant preference. Before the interview, participants were asked to complete questionnaires on demographic and medical characteristics. Interviews were facilitated by a trained study staff who followed a semi‐structured guide informed by Fitch's [25] Supportive Care Framework to elicit open discussion on key domains of supportive care needs. Topics included information and support received at diagnosis and after treatment (e.g., “What did you think about the amount of information you received?”; “What topics did you want information on then/or still want now?”; “What are some medical or support services that you wanted then/or still want now?”), needs as a PaCa survivor (e.g., “What are your main needs and concerns as a PaCa survivor?”; “What has it been like getting your needs met?”), and health behaviors (e.g., “What has your health been like since completing your PaCa treatment?”; “How have you addressed your symptoms after your PaCa treatment?“). Interviews lasted approximately 2 h, were audio recorded and transcribed, and participants were compensated $50. Participants were assigned a unique identifier not associated with personal identifying information. Interviews conducted in Spanish were professionally translated to English by SCCC's Behavioral and Community‐Based Research Shared Resource (BCSR).

### 2.3 Data Analysis

Demographic and medical characteristics were analyzed with descriptive statistics. We applied Rapid Qualitative Analysis (RQA) to analyze the one‐on‐one interviews following Watkins' guidelines [29]. RQA can be a more efficient approach than traditional qualitative analysis; it involves team members individually summarizing key points from qualitative data and then combining individual data by categorizing related key points to produce relevant themes [30]. Four team members (S.E.F.‐P., R.G., A.N., and A.E.B.) with psycho‐oncology experience were trained on RQA. Each team member independently summarized key information from interview transcripts, coded the transcripts using a structured matrix, and collaborated to produce a comprehensive final matrix summarizing data to facilitate the identification of themes. The team met to discuss and reconcile any discrepancies, ensuring consistency and reliability in theme identification. Disagreements were resolved though consensus, with a senior qualitative researcher (R.G.) making the final determination when needed.

### 2.4 Ethical Considerations

Ethical approval was granted by UM's Institutional Review Board (eProst #20190846). Informed consent was obtained from all study participants.

## 3 Results

### 3.1 Participant Characteristics

On average, participants (*N* = 18) were 64.2 years old (SD = 9.4), 66.7% were female, 88.9% were White, 55.6% identified as Hispanic/Latino, 50% graduated with a 4‐year college degree or above, 61% were married, and 33.3% completed the interview in Spanish. Most participants underwent a Whipple procedure (72.2%), with smaller proportions receiving a distal pancreatectomy (22.2%) or no surgical intervention (5.6%). Additionally, 83.3% of survivors received neoadjuvant chemotherapy. While cancer staging information was collected, a large proportion (66.7%) had unknown staging due to variability in medical record documentation and self‐report limitations. Table 1 presents demographic and medical characteristics.

**TABLE 1:** Participant demographics and medical characteristics.

Demographics and medical characteristics	Total sample (*n* = 18)
Age	*M* = 64.2; SD = 9.4
Gender	*n* (%)
Female	12 (66.7)
Male	6 (33.3)
Race	
White/Caucasian (including White Hispanic)	16 (88.9)
Black/African American or Afro‐Caribbean Black	2 (11.1)
Ethnicity	
Hispanic	10 (55.6)
Non‐Hispanic	8 (44.4)
Employment status	
Full‐time	2 (11.1)
Part‐time	2 (11.1)
Unemployed with disability	4 (22.2)
Unemployed without disability	1 (5.6)
Retired	7 (38.8)
Student	1 (5.6)
Unknown	1 (5.6)
Education	
High school/GED	3 (16.7)
Some college	6 (33.3)
Graduated 4‐year college or above	9 (50.0)
Marital status	
Married	11 (61.0)
Divorced/separated	3 (16.7)
Single/never married	3 (16.7)
Widowed	1 (5.6)
Cancer stage	
Stage I	0 (0)
Stage II	2 (11.1)
Stage III	2 (11.1)
Stage IV	2 (11.1)
Unknown	12 (66.7)
Surgical procedure	
Whipple procedure (pancreaticoduodenectomy)	13 (72.2)
Distal pancreatectomy	4 (22.2)
No surgical procedure	1 (5.6)
Neoadjuvant chemotherapy	15 (83.3)

### 3.2 Qualitative Themes

**FIGURE 1:**
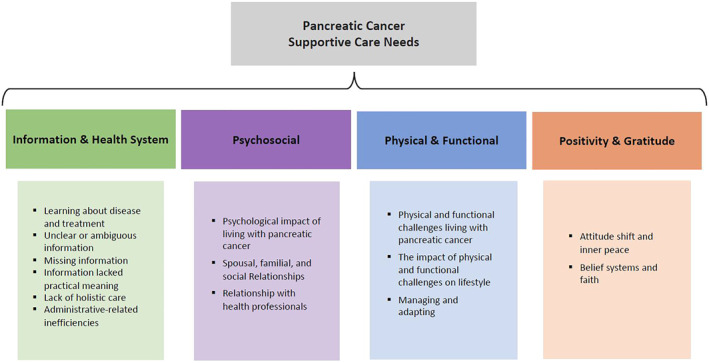
Illustration of key themes.

Three major themes related to supportive care needs were identified: (1) information and health system needs, (2) psychosocial needs, and (3) physical and functional needs. One additional theme emerged regarding the experiences of living with PaCa: positivity and gratitude. Each is discussed in turn below. Figure 1 presents key themes. Table 2 presents illustrative quotes.

**TABLE 2:** Illustrative quotes extracted from transcripts.

Health system and information needs (diagnosis and follow‐up)
*Learning about and comprehending disease, treatment, and support*
“Amount of information was enough; not too much where it's overwhelming to digest and listen to.”
“My son works in the health field, at home he always told me if I had any doubts about something, it was much easier to understand. When something different happened, he would tell me to let him know.”
“No trouble comprehending information because it was given little by little as the process was going, which made it easier to understand.”
*Unclear or ambiguous information*
“Yeah. Yeah. They don't call it remission. They say I'm cancer free, period. So, that's … And I don't know what constitutes remission or being cancer free, I don't know what the difference is but I would think that it's remission until so many years have gone by. I don't know, they use those terms. Do they interchangeably use them sometimes, sometimes they don't, I don't know.”
“I'm getting scans every 3 months and I'm getting bloodwork every 6 weeks because my tumor numbers were a little bit elevated until the doctor had poked around, and he did that Notero blood test that they do. They come to the house to do it. Then they ship it away to some special lab, I don't know. And that's when they give me some kind of read out. I don't know what's going on but a doctor's visit, you know … It's technical and I'm not in the medical field.”
*Missing information*
“They didn't explain it was the neuropathy. That is the major thing you get, so that has to be put in the forefront, not like a maybe you'll get it. Everybody that gets that type of Folfirinox will get neuropathy. It's the extent of neuropathy you have to be careful with.”
*Information provided lacked practical meaning*
“Mainly the question of side effects, that they would tell us what they are because they always manage a big list of symptoms and say, “they may or may not happen to you.” I understand that but that's very ambiguous, that doesn't help a patient, it doesn't help to say a list of 50 things that may or may not happen. That doesn't help emotionally.”
“I do think that it would have been nice to have had that additional support that I had asked for because it's not all about the medical information. A lot of it is, how is this gonna impact my life? How should I, how can I cope with this, et cetera, et cetera? And that was lacking completely. There was no face to those questions for me. It just wasn't there.”
“Sometimes doctors will just tell you what you need to know, not what—you know, what they want you to know, not exactly what you need to know. And with a serious disease like this, you have to know what you're getting into.”
“Doctors give you the medicine but they do not take it, they only talk about what the literature says, but they do not experience the physical discomfort that chemotherapy causes in the face of any type of cancer, in my case, pancreatic cancer.”
*Lack of holistic care within health system*
“So, the other thing about when you get caught up with the cancer, don't take a victory lap, because the patient has other needs.”
“They said not to worry about anything else and so I have not. Obviously, there's fears of my own, that's a personal fear that it could come back but they tell me I'm doing really great, and they don't foresee anything like that. Of course, it could always happen because cancer has a way of doing that, but they think that they did well, especially since they were able to take enough of … you know, for markers, they had a clean marker so that everything was good.”
“Baptist did not have a hands‐on approach of how to take care of a whole person. They were just dealing with the disease. Sylvester is different. They handled the whole thing. They talked to me about how I was gonna lose my hair and that I was able to get a wig if I needed to. I Opted to just go bald. They offered that if I needed a counselor, I didn't feel that I needed it.”
*Administrative‐related inefficiencies*
“It's frustrating to need something, and it's gonna take 6 months to get an appointment with inpatient rehab.”
Don't know him that well, so it's better that you call him and keep on calling him until you get through.”

Psychosocial needs (affective and social aspects)
*Psychological impact of living with pancreatic cancer*
“It's very important because a person tends to be depressed, ‘I am going to die,’ no, another way.”
“Fear of getting sick again and not knowing what the next steps are, resulting in anxiety of what tomorrow brings, making the changes difficult.”
“Concerns of recurrence.”
“Main concern is that you're gonna have recurrence”
“Psychological, as in grief of losing your previous lifestyle.”
“I believe that I have decided to die with dignity, that is what it is all about. I understand my condition, and I know there is no cure, but I have decided to die with dignity, I am not talking about living, I am talking about dying; in my case, it is to die with dignity.”
*Spousal, familial, and social relationships*
“The most difficult part is being sick and being able to adapt, but it's hard to change the life of those around you to adapt as well.”
“And it is—it was definitely scary for my family, so I feel like the people who needed—it was more scary for them than for me, I think. And I feel like if anything, it's the people surrounding the person who's been diagnosed who need the help, who need someone to talk to, who need someone to calm them down and help them through it, more than the actual person themselves.”
“When we went through the whole treatment, I was fortunate to have my family by my side, sharing with everyone the treatment; they all knew what it was about, they all knew what I was doing; they took turns to accompany me to the appointments. That was very important because I was not alone.”
“My friends bringing meals to us. When I say friends, my church family for the most part. Not just bringing meals, but staying, and lifting me up, and making me laugh, coming to the hospital when I was in the hospital, coming to rehab when I was in rehab, coming to my home. So many things.”
“And my bosses were so good, they let me work from home. They set me up with an office at home, everything. And they don't expect me to come back. I can stay home.”
“I know I talked to a lot of—I talk to people who have been diagnosed and sometimes when they're just diagnosed they need to just talk to somebody who's been through it. And I feel like that's something that's a real positive for people who are diagnosed because it is such a scary diagnosis.”
“PanCan, you know. We're on that because there's a lot of good information that you see from pancreatic cancer survivors which is similar to what you're talking about. It's great to be able to be able to look at these things and understand.”
*Relationship with health professionals*
“And there were certain emotional needs that I was asking for that were neglected completely … I Chalk it up to that. I Chalk it up to people who've been working in the medical field for so many years, I think they might become a little bit—I don't want to be negative—I'm not sure what the word is that I want to use, but they're so used to dealing with patients that they forget or de‐emphasize the emotional aspect.”
“I was in a hallway being taken care of … He came in and he was very straightforward. He said this is bad. I don't see—this is not a good sign. We're gonna do everything we can to help you but you probably should go home and prepare for the worst. I Expect you … 15% chance to be here next year.”

Physical and functioning needs
*Physical and functional challenges living with pancreatic cancer*
“And because of my back pains, I couldn't do lifting. I couldn't do things—back and neck pains. So, my house turned into a nightmare.”
“I must mention that there are after‐effects of the chemotherapy treatment; I have neuropathy in my feet, that will not go away… I have had it for 3 years, and if I live longer it will not go away.”
“… they took out two thirds of my stomach and I can only eat eight ounces less than eight ounces at a time.”
“That the chemo makes many people sensitive to cold, and I was one of those many people. And just drinking a cold drink of water was very uncomfortable… anything with any cold temperature was like fire.”
“I got diabetes 1 because my pancreas is half of what it was because of the surgery, I don't have gall bladder or appendix either, so I take medication to assimilate the food because if I don't take it I feel sick to my stomach … Every time I eat or have breakfast, whatever it is, I have to take it. That is from now on.”
*The impact of physical and functional challenges on lifestyle*
Less mobility
“Not being able to travel as much”
“Unable to do long car trips physically”
Less flexibility
“Unable to plan in advance for outings or vacations because you never know when your next treatment will be scheduled for; having to cancel plans often due to treatment schedule.”
Financial sacrifices
“Financial sacrifices, like going from a full salary to 60% of it due to being on disability, and not being able to make extra money.”
*Managing and adapting*
“Being more cognizant of what I eat.”
“In my case it was, to take care of the fat a little bit, because excess is not good for anyone, so I try to eat as healthy as I can, always including vegetables and salad in my daily meals.”
“To stay healthy during chemo, I worked out, I ate properly, gave up all vices, tried to stay in with a normal weight. I'm unique because I have a hard time losing weight. I did have a time of gaining weight”
“I don't go out late and eat like I used to. I don't drink like I used to. Socially, but still, I don't drink. I don't travel far. I Mean it's just something that I still have to—and I'm working on it, you know? … I will go out and have a cocktail if … you know, one time in a month maybe but it's not something—we used to have wine with all our dinners, you know? We don't do that anymore. Those kind of things.”
“… I don't have two thirds of my stomach and I'm replumbed and I need somebody to really look at how can we structure what this person is eating. I've done it myself and I think I'm doing a pretty good job of it, but it's been definitely trial and error.”
Rationale for adopting a healthy lifestyle
“I cannot eat beans, I cannot eat some vegetables, a lot of things. Not because the doctors said to me, it's because I am seeing how my body reacts to those foods.”
“It's a lifelong journey. I think about this often. It's not a … oh, I beat it. You don't beat it. You work at it. It's a job and you gotta keep working at it and make sure that you do everything humanly possible to try and overcome this. Not overcome it but to live with it.”

Positivity and gratitude
*Attitude shift and inner peace*
“Being more appreciative of life and not taking as many things for granted like friends and family, nature, etc.”
“…That a diagnosis does not mean that you are going to die immediately if you look at it positively. Here I am, I was diagnosed 3 years ago, and with surgery; always thinking positively, is important.”
“All I was thinking about was getting well. It just kind of nullifies that everything is gonna be fine, the doctor's gonna be successful, and that the chemo would be successful. All the sickness that comes with it was going to work, you know. I just felt if I kept thinking positive, positive, positive, it was going to be better, and most people didn't.”
*Belief systems and faith*
“This is my destiny; this is my fate. I have to pass by all of this and be a winner of this battle.”
“The Lord has been with me every step of the way, and I have felt as I would go through things, my story, which we have just touched the tip of the iceberg, but my story of what they would tell me to expect and then that didn't happen at all, it was just blessing on top of blessing on top of blessing.”

#### 3.2.1 Theme 1. Information and Health System Needs (Diagnosis and Follow‐Up)

##### 3.2.1.1 Learning About Pancreatic Cancer and Treatment

Learning about PaCa was crucial for participants, who gathered information from the internet (e.g., hospital websites, government websites), healthcare providers, and cancer survivors. At diagnosis, participants received information on their cancer severity and survival rate, treatment options and recommendations, potential treatment side effects (e.g., loss of appetite, nausea, diarrhea), complementary therapies (e.g., acupuncture), as well as support groups like Pancreatic Cancer Action Network (PANCAN) and Gilda's Club, a community‐based organization offering emotional and social support for cancer survivors, friends, and families. Participants reported receiving a moderate amount of information that was “*just enough without feeling overwhelmed*.” Participants with a family history of cancer found it easier to comprehend cancer‐related information. For example, one participant stated, “*My mom had cancer, my dad had cancer, I had to go through it with them, so I knew more or less*.” Another participant explained, “*I understood it very well, perhaps because I already had the background of my father's condition.*”

Following treatment, participants reported receiving comprehensive information covering various aspects such as treatment results, follow‐up tests, medications, treatment side effects, recovery length, potential cancer recurrence, and rehabilitation services. Additionally, they were provided information on dietary considerations, maintaining physical activity, and managing stress. Participants were satisfied with post‐treatment information, feeling well‐informed. They appreciated the gradual delivery of information, which aided understanding, and some benefited from family members in healthcare for grasping complex concepts.

##### 3.2.1.2 Information Needs

Participants reported receiving unclear or ambiguous information, particularly regarding certain medical concepts and lab results, which resulted in difficulties understanding. For instance, some participants described uncertainty about “*what constitutes remission or being cancer free*,” while others found that the laboratory results were “*too technical to understand*.” Furthermore, several participants expressed concerns about missing information, particularly emphasizing the need for details regarding post‐chemotherapy neuropathy, post‐treatment procedures, and specific details about the areas of organ removal due to surgery. In one case, a participant described a significant consequence resulting from the unintended use of an oil‐based pain reliever. The participant had not been informed that the Whipple surgery had removed his gallbladder, a crucial organ for processing oil. Another participant expressed deep apprehension: “*What happens next if I fail these tests? What's my alternative? Do I have to go back to this or nobody tells you*.”

Participants found the information provided overly lengthy and lacking in practical application. While they understood the content, they struggled with how to apply it, especially regarding chemotherapy side effects. One participant noted, “*I remember reading it on the chart but …not being very specific that these things really can happen*,” leading to feelings of surprise and unpreparedness when symptoms occurred. Some suggested a “patient portal” to access specific information about chemotherapy side effects. Similarly, participants found the nutrition information extensive but impractical, as one described: “*It did explain everything, but you're kind of still left with… yeah, the how do I do that*.” This feedback emphasized the need for more actionable and relevant guidance.

##### 3.2.1.3 Health System Needs

Participants expressed concerns about the limited integration of holistic care within the health system. They observed that the system emphasizes medical outcomes (such as being “cancer‐free”) while emotional needs remained unmet. For example, there was a noted absence of follow‐up care, leading to uncertainty about the next steps if the initial treatment was unsuccessful. Participants emphasized the importance of a care approach that addresses their overall well‐being, rather than focusing solely on the disease outcome.

##### 3.2.1.4 Administrative‐Related Inefficiencies

Some participants mentioned a lack of awareness of available support services: “*There's a huge gap in people knowing about the services provided for them*.” Other participants experienced long wait times (e.g., *six months to get an appointment with inpatient rehab*) and encountered difficulties with scheduling follow‐up medical appointments.

#### 3.2.2 Psychosocial Needs

##### 3.2.2.1 Psychological Impact of Living With Pancreatic Cancer

Participants frequently reported psychological challenges and a strong need for emotional support from diagnosis to follow‐up, with some feeling their emotional needs were not fully addressed at diagnosis. They emphasized the need for a more holistic approach to care, considering both medical and emotional aspects. After treatment, many struggled with chemotherapy side effects, fear of recurrence, and regaining strength, highlighting the importance of mental health support. Participants also expressed feelings of isolation, depression, and grief over losing their previous lifestyle, with a lack of psychological support for coping. One participant reflected, “*Physically, I'm good. All the tests came in negative… I've taken all those. And clean. But it's the mental health. What's gonna happen next? The unsureness. But they should maybe offer that. It's not just pulse treatment; you're done. You don't get off chemo; you're done. It doesn't work that way. It's five, 10 years down the road.*”

Despite psychological challenges, participants expressed a desire to maintain a quality of life beyond mere survival. Many discussed the importance of regaining strength and *maintaining an active social life*, aiming for a sense of normalcy and purpose in their daily lives.

##### 3.2.2.2 Spousal, Familial, and Social Relationships

Participants shared a common need for guidance on effectively communicating their diagnosis and treatment journey with those in their close circle. They wanted support in navigating difficult conversations with loved ones, family, and friends. Specifically, they described the need for assistance in processing their emotions while also managing the expectations and reactions of those around them. Participants reported that adjusting the lives of those around them was difficult, as they dealt with the fear and anxiety of potential cancer recurrence coupled with uncertainty about the future. Several participants were concerned about the health and stress levels of their caregivers. They emphasized the importance of making psychological counseling available not only for themselves but also for their caregivers.

Overall, participants acknowledged that the support received from spouses, family, friends, church, or colleagues made lifestyle changes easier. Most viewed family support as a primary need. Participants generally expressed being more compassionate and thoughtful toward themselves and others. It should not be overlooked that some participants found communicating with other cancer survivors (e.g., PaCa support groups) helpful, recognizing it as a way to share experiences, acquire knowledge, and gain understanding.

##### 3.2.2.3 Relationships With Health Professionals

While many participants appreciated the support and trust from healthcare professionals, some felt their emotional needs were not fully addressed, especially during diagnosis and follow‐up. A few noted that communication could have been clearer and more factual, rather than subjective or negative, which left them feeling unsupported and stressed. Participants emphasized the need for compassionate, empathetic communication and reported that unanswered concerns about follow‐up care contributed to anxiety and uncertainty, particularly around unexpected tests or potential treatment outcomes.

#### 3.2.3 Physical and Functional Needs

##### 3.2.3.1 Physical and Functional Challenges of Living With Pancreatic Cancer

Participants reported a range of physical and functional challenges during and after treatment, including limited energy levels, post‐chemotherapy neuropathy (e.g., *tingly hands*, *difficult standing*), heightened sensitivity to cold temperatures (e.g., “*anything with any cold temperature was like fire*”), fluctuations in weight (e.g., “*the more try to diet and get it off, the more it seems to come back on*”), and enduring post‐surgery pain (e.g., “*couldn't do things—back and neck pains*”). Furthermore, many participants encountered difficulties related to food intake, such as changes in their sense of taste and digestion issues. A few participants even developed diabetes, requiring them to take medication.

In addition, participants brought up unexpected treatment‐related sexual issues, expressing shock and a need for support in addressing these challenges: “*And one thing they never mention at all is your sexual appetite or what will happen to you down there. And I was a tad surprised because it's been a 180 completely for me. It's like a different life altogether.*”

##### 3.2.3.2 The Impact of Physical and Functional Challenges on Lifestyle

Participants faced significant lifestyle changes due to physical and functional challenges. They experienced a decline in mobility, including limitations such as reduced travel opportunities and the inability to walk unaided. Some even relocated family for support during treatment. Participants also described their decreased ability to engage in hobbies due to their limited mobility capability: “*There are some things now that I've had to give up, like scuba diving. I just physically can't do it anymore*.” The inflexibility imposed by treatment schedules added a layer of difficulty, with participants reporting it “*difficult to make lifestyle changes during treatment because chemo and appointments take up time*.” Unpredictable medical appointments and chemotherapy treatments forced frequent plan cancellations, disrupting social activities and personal routines. Furthermore, participants reported making financial sacrifices as they were compelled to work fewer hours due to their limited physical and functional abilities.

##### 3.2.3.3 Managing and Adapting

Despite the negative impact of those physical and functional challenges, participants described their efforts to maintain a healthy lifestyle. They made conscious efforts to manage their food and beverage intake, including reading nutrition labels and prioritizing vegetables over sugary foods. Participants also emphasized the importance of adhering to strict mealtime routines, reducing alcohol consumption, quitting smoking, and controlling their weight with engagement in exercise. However, it is important to highlight that some participants expressed the need for clearer instructions regarding post‐treatment rehabilitation, as they often “*underestimate how much exercise is needed for rehab after treatment*,” as well as nutrition.

#### 3.2.4 Positivity and Gratitude

##### 3.2.4.1 Attitude Shift and Inner Peace

It is important to recognize that participants emphasized positivity and gratitude. They described having developed a greater appreciation for life, no longer taking things like relationships with friends and family and the beauty of nature for granted. Notably, participants reported experiencing a shift in attitudes, becoming more positive and thoughtful toward themselves and others. For instance, participants expressed a strong willingness to share their journey living with PaCa, hoping to assist future patients and their families “*to understand and to see it positively*.”

##### 3.2.4.2 Belief Systems and Faith

Participants shared their religious beliefs and perspectives on their cancer journey, which served as a source of comfort and guidance during challenging times.

## 4 Discussion

### 4.1 Main Findings

The current study identified specific supportive care needs within a racially and ethnically diverse population of PaCa survivors in Miami, with four major themes: information and health system needs, psychosocial needs, physical and functional needs, and an emerging theme of positivity and gratitude. Participants emphasized the importance of understanding their disease and treatment options and seeking information from various sources like healthcare providers and support groups. While many felt adequately informed, some struggled post‐treatment with understanding medical concepts and lab results, particularly regarding chemotherapy side effects and nutritional guidance. Participants called for a more holistic approach, addressing emotional needs and integrating care such as psychotherapy and nutritional counseling. Administrative issues, like long wait times and lack of support service promotion, were also highlighted as areas for improvement.

Psychosocial needs were significant, with patients expressing feelings of isolation, fear of cancer recurrence, and grief over losing their previous lifestyle. They emphasized the need for mental health support, including stress management and coping skills to manage the emotional challenges of living with PaCa. Patients also expressed the importance of maintaining quality of life and the dignity in facing death.

In terms of relationships, patients highlighted the difficulty of communicating their diagnosis and treatment experiences to loved ones, expressing a need for guidance in handling these sensitive conversations. They also noted the importance of psychological counseling for caregivers, recognizing the significant stress and anxiety their condition placed on their families. Support from spouses, family, friends, and other cancer survivors was deemed crucial, though some patients struggled with accepting help.

Relationships with health professionals varied among patients. While many appreciated the support and trust established with their healthcare providers, some felt that their emotional needs were not fully addressed, especially at the time of diagnosis and during follow‐up care. A few patients mentioned experiencing communication challenges, such as a perceived lack of empathy or the use of unclear language. Overall, patients desired more compassionate communication and clearer guidance regarding their treatment and follow‐up care.

Participants reported significant physical and functional challenges during and after treatment, including low energy, neuropathy, cold sensitivity, weight fluctuations, and persistent pain. Issues with food intake, altered digestion, and developing diabetes requiring medication management were common. Unexpected sexual side effects from chemotherapy, not previously discussed, also caused distress. These limitations affected mobility and daily activities and led to lifestyle adjustments, including relocating for family support. Unpredictable treatment schedules and financial strain from reduced work added further stress. Despite these challenges, patients focused on maintaining a healthy lifestyle while expressing the need for clearer guidance on post‐treatment rehabilitation and nutrition.

Although participants expressed various unmet needs throughout their journey, they also reported experiencing a significant shift in their attitudes, characterized by increased positivity and gratitude. They described developing a deeper appreciation for life, valuing relationships, and finding joy in the simple beauty of nature. This shift in mindset led them to become more thoughtful toward themselves and others, with many expressing a desire to share their experiences to help future patients view their journey more positively. Additionally, patients found comfort and guidance in their religious beliefs, viewing their cancer journey as part of their destiny and drawing strength from their faith to navigate the challenges they faced.

While our study aimed to explore supportive care needs in racially and ethnically diverse PaCa survivors, the current findings reflect common themes across all participants rather than subgroup‐specific differences. Nonetheless, existing literature suggests that historically underrepresented populations with PaCa may face unique challenges, including disparities in access to treatment, language barriers, and culturally distinct coping mechanisms [28, 31]. Some of our participants, particularly Spanish‐speaking individuals, expressed difficulties in understanding complex medical information and accessing culturally tailored supportive services. Future studies should examine how racial and ethnic factors shape supportive care needs.

### 4.2 Clinical Implications

The findings highlight several key clinical implications for improving supportive care for PaCa survivors. Participants expressed challenges in understanding complex medical information and navigating the healthcare system, suggesting a need for enhanced educational resources, including multilingual patient portals, culturally tailored materials, and structured survivorship care plans. Psychosocial support is another key area for intervention. Given the levels of anxiety, fear of recurrence, social isolation, and emotional distress reported by participants, a more holistic approach addressing emotional and psychological needs, with integrated psychosocial support, is essential. This may involve integrating mental health specialists within cancer treatment centers and offering routine psychosocial screenings for survivors. Additionally, expanding access to peer support groups and survivorship counseling could help address patients' emotional needs more comprehensively. From a functional and lifestyle perspective, structured post‐treatment rehabilitation programs should be developed to assist survivors in managing long‐term side effects. Our findings underscore the importance of specialized nutritional counseling and guidance on physical activity to help survivors navigate post‐treatment lifestyle adjustments.

### 4.3 Study Strengths and Limitations

The study's strengths include its qualitative design, which allowed for an in‐depth exploration of the lived experiences of PaCa survivors, and the thematic analysis that provided a comprehensive understanding of their diverse needs and challenges. The inclusion of Hispanic participants (55.6%), with over one‐third completing interviews in Spanish, offers valuable insights into a historically underrepresented ethnic group in PaCa research. However, the sample was predominantly White (88.9%), with limited representation of Black/African American (11.1%) participants, which may impact the generalizability of the findings to broader populations. Additionally, the study has limitations such as potential recall bias due to reliance on experiences that occurred during a potentially traumatic time, a sample size that may not capture all possible experiences and perspectives, and findings that may not be generalizable to all PaCa survivors, particularly those from cultural or socioeconomic backgrounds that differ from Miami's population.

### 4.4 Conclusions

This study confirms the complex supportive care needs of PaCa patients, requiring a comprehensive approach that includes clear communication, holistic care, caregiver support, and structured rehabilitation. Emotional and psychological support is essential for enhancing patient well‐being. Future research should prioritize developing psychosocial interventions to address these needs and improve the quality of life for PaCa survivors.

## Author Contributions

Frank J. Penedo, Nipun B. Merchant, Roberto M. Benzo, and Sara E. Fleszar‐Pavlovic contributed to the study conception and design. Material preparation, data collection and analysis were performed by Sara E. Fleszar‐Pavlovic, Roberto M. Benzo, Rui Gong, Amber Browder, and Aria Nawab. The first draft of the manuscript was written by Sara E. Fleszar‐Pavlovic, Rui Gong, Amber Browder, and Aria Nawab. All authors commented on previous versions of the manuscript. All authors read and approved the final manuscript.

## Ethics Statement

This study was performed in line with the principles of the Declaration of Helsinki. Approval was granted by University of Miami's Institutional Review Board (ePROST# 20190846 on 12/16/2019).

## Consent

Informed consent was obtained from all individual participants included in the study.

## Conflicts of Interest

The authors declare no conflicts of interest.

## Data Availability

The data that support the study findings are available from the corresponding author upon request. Access to data is restricted due to patient privacy.
